# Case Report: Local Anesthesia Round Window Plugging and Simultaneous Vibrant Soundbridge Implant for Superior Semicircular Canal Dehiscence

**DOI:** 10.3389/fneur.2020.581783

**Published:** 2020-12-22

**Authors:** Giulia Mignacco, Lorenzo Salerni, Ilaria Bindi, Giovanni Monciatti, Alfonso Cerase, Marco Mandalà

**Affiliations:** ^1^Otolaryngology Unit, Department of Medicine, Surgery and Neuroscience, University of Siena, Siena, Italy; ^2^Neuroimaging, Diagnostic and Functional Neuroradiology Unit, Department of Neurological and Movement Sciences, University of Siena, Siena, Italy

**Keywords:** superior semicircular canal dehiscence, round window plugging, round window reinforcement, middle ear implant, canal dehiscence syndrome

## Abstract

The aim of the present study is to report the outcomes of round window reinforcement surgery performed with the application of a Vibrant Soundbridge middle ear implant (VSB; MED-EL) in a patient with superior semicircular canal dehiscence (SSCD) who presented with recurrent vertigo, Tullio phenomenon, Hennebert's sign, bone conduction hypersensitivity, and bilateral moderate to severe mixed hearing loss. Vestibular evoked myogenic potentials (VEMPs) and high-resolution computed tomography (HRCT) confirmed bilateral superior semicircular canal dehiscence while this was not seen in magnetic resonance imaging. The surgical procedure was performed in the right ear as it had worse vestibular and auditory symptoms, a poorer hearing threshold, and greatly altered HRCT and VEMPs findings. With local-assisted anesthesia, round window reinforcement surgery (plugging) with perichondrium was performed with simultaneous positioning of a VSB on the round window niche. At the one and 3 months follow-up after surgery, VSB-aided hearing threshold in the right ear improved to mild, and loud sounds did not elicit either dizziness or pain in the patient.

## Introduction

Superior semicircular canal dehiscence (SSCD) was first described by Minor et al. (1), and is characterized by a number of peculiar audio vestibular signs and symptoms (1). Common symptoms are autophony and hyperacusis, aural fullness, dizziness or vertigo/nystagmus induced by intense noises (Tullio phenomenon), or pressure via pneumatic otoscopy (Hennebert's sign). Audiometric findings can include both an air-bone gap or mixed type hearing loss, and/or suprathreshold bone scores. Typical signs and symptoms are secondary to a third window syndrome that results from a dehiscent superior semicircular canal (2).

Both high resolution computed tomography (HRCT) with reconstruction on the plane of the superior canal and vestibular evoked myogenic potentials (VEMPs) are mandatory to confirm the diagnosis of SSCD.

Surgical approaches are not mandatory in SSCD. The decision to treat the pathological canal side is based on various assessments including severity of symptoms, surgical candidacy of patients, and their desire for an improvement in quality of life. Conventional surgery provides direct treatment of the canal fistula through either a transmastoid or middle cranial fossa approach: the most common and well-described surgical treatments include capping, resurfacing, and plugging of the superior semicircular canal (3, 4).

Despite the demonstrated efficacy, these interventions are invasive and imply a potential risk of persistent hearing deterioration and vestibular loss. Both the middle fossa and transmastoid approaches require general anesthesia which might increase the overall surgical and anesthesiologic risk. Recently, it has been proposed that a two-window inner ear system can be restored by directly plugging the round window (RW). This procedure has been demonstrated to be safe, fast, and effective compared to classical SSCD surgical treatments (5, 6).

The Vibrant Soundbridge middle ear implant (VSB; MED-EL, Innsbruck, Austria) is an implantable hearing aid that transduces sounds into electromechanical vibrations to the ossicular chain or directly to the RW. It is indicated for the treatment of conductive or mixed moderate to severe hearing loss (7).

We describe the first case in literature of RW plugging and VSB positioning performed simultaneously under local anesthesia in a patient with bilateral SSCD and severe mixed hearing loss.

## Case Description

A 78-year-old woman was referred to our clinic with bilateral hearing loss with sound distortion, tinnitus and auditory hypersensitivity, recurrent vertigo/dizziness induced by loud noises (Tullio phenomenon), and a diagnosis of bilateral SSCD at the temporal bone HRCT performed in the emergency room (**Figures 2a–c**).

**Figure 1:**
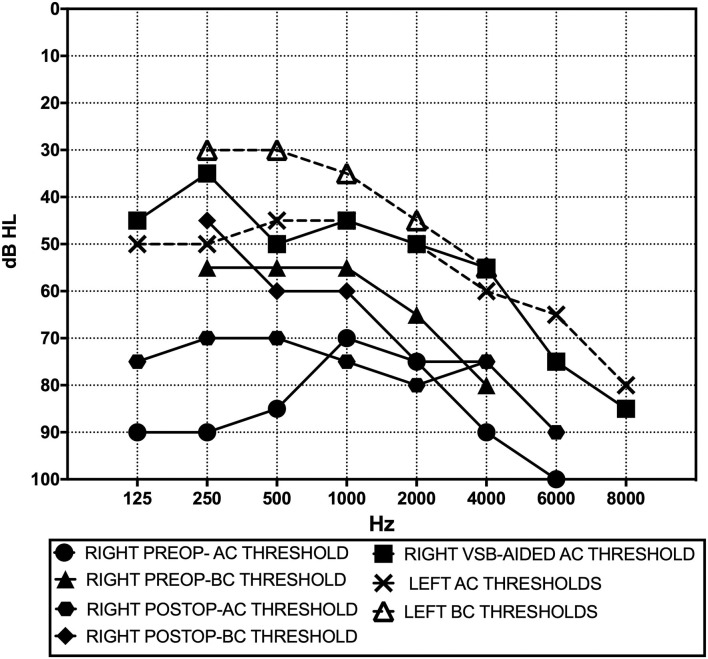
Pre- and postoperative (3-month) pure tone audiograms.

**Figure 2:**
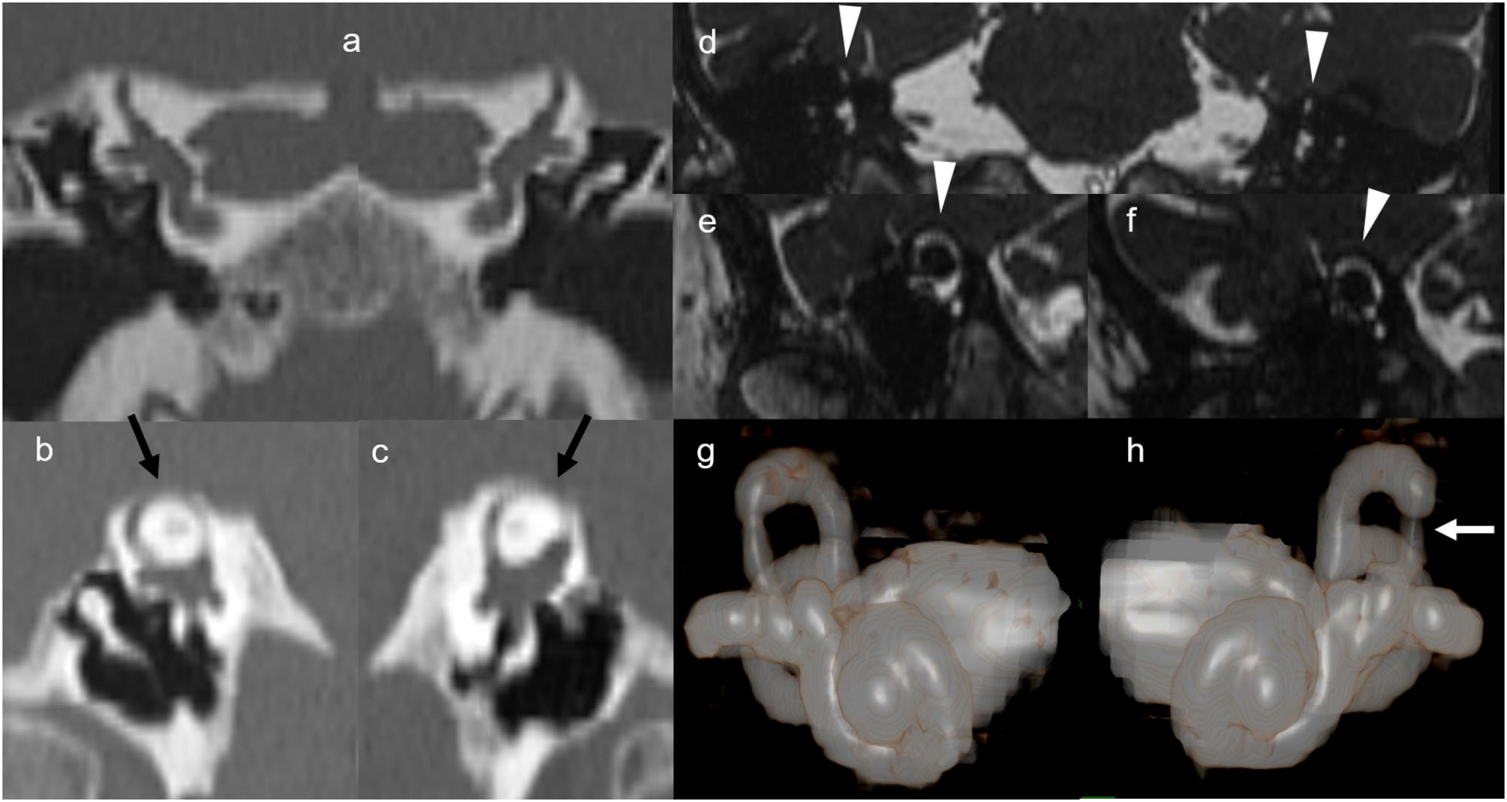
High-resolution computed tomography and magnetic resonance imaging. Multidetector computed tomography 1.0 mm-collimated coronal **(a)**, and 0.5-mm collimated right **(b)** and left **(c)** sagittal oblique (so called Poschl plane) reformatted images obtained at admission in the emergency unit. Images according to the Poschl plane clearly show 5.5 and 3.5 mm-wide dehiscence of the bone overlying the right and the left superior semicircular canals, respectively (black arrows). These findings are consistent with clinical and audiometry findings. 1.5T magnetic resonance 3D-true fast imaging with steady-state free precession coronal **(d)**, right **(e)**, and left **(f)** sagittal oblique reformatted images obtained 2 months before as outpatient did not show the dehiscence (arrowheads). Note that right **(g)** and left **(h)** magnetic resonance 3D volume rendering anterior views shows thinning of both the lateral crus of both the superior semicircular canals, mainly in the left side (white arrow).

Preoperative audiometry indicated severe mixed hearing loss in the right ear, with moderate conductive hearing impairment in the left ear (Figure 1). A pure tone sound of 110 dB at 500 and 1,000 Hz in the right ear, and pneumatically increasing external auditory canal pressure induced dizziness without detectable nystagmus on video-oculographic (or Frenzel goggles) examination. Her tympanogram was bilaterally normal. A stapedial reflex could not be performed due to patient intolerance (dizziness). Mastoid vibration elicited dizziness without detectable nystagmus on video-oculographic (or Frenzel goggles) examination. A temporal bone 1.5-T MRI with 3D reconstruction performed two months before did not show the bilateral dehiscence (Figures 2d–h). Air conduction cervical VEMPs (cVEMPs) demonstrated a threshold of 85 dB HL on the right side and 100 dB HL on the left side. The cVEMPs were recorded from both ears using 500 Hz short tone-bursts (STBs). A video head impulse test for horizontal and vertical canals, including both dehiscent SSC, showed normal vestibulo-ocular reflex gain bilaterally (Table 1).

**Table 1:** Pre- and post-operative evaluation steps.

T^0^	•Pure tone audiometry: R: severe mixed hearing loss; L: moderate conductive hearing loss • Pure tone audiometry: R: severe mixed hearing loss; L: moderate conductive hearing loss • Air conduction VEMPs: R: threshold of 85 dB HL; L: 100 dB HL • VHIT: normal vestibulo-ocular reflex gain bilaterally • Mastoid vibration: dizziness without Ny
T^1^	Radiological assessment: temporal bone 1.5T MRI with 3D reconstruction + temporal bone high resolution multidetector CT
T^2^	Round window plugging and simultaneous VSB on right ear
T^3^	1-month post-operative follow-up: VSB activation and hearing and vestibular evaluation
T^4^	3-months post-operative follow-up: hearing and vestibular evaluation
T^5^	6-months post-operative follow-up: hearing and vestibular evaluation

*R, right; L, Left; VHIT, video head impulse test; Ny, nystagmus; MRI, magnetic resonance imaging; CT, computerized tomography; VSB, vibrant sound bridge*.

*T^0^, beginning of the evaluation; T^1−6^, next evaluations*.

## Diagnostic Assessment and Therapeutic Intervention

Possible surgical procedures (superior canal plugging or resurfacing either through a middle fossa approach or via a transmastoid route, or RW niche plugging) were discussed with the patient. Comorbid cardiopulmonary conditions represented major contraindications for general anesthesia, so the latter procedure was the only possible option to pursue.

Since the patient did not show bilaterally any benefit from an air conduction hearing aid but rather had deteriorating auditory and vestibular symptoms on the right, we decided to perform the surgical procedure in the right ear as it had worse vestibular and auditory symptoms, a poorer hearing threshold, and greatly altered HRCT and VEMPs findings.

**Figure 3:**
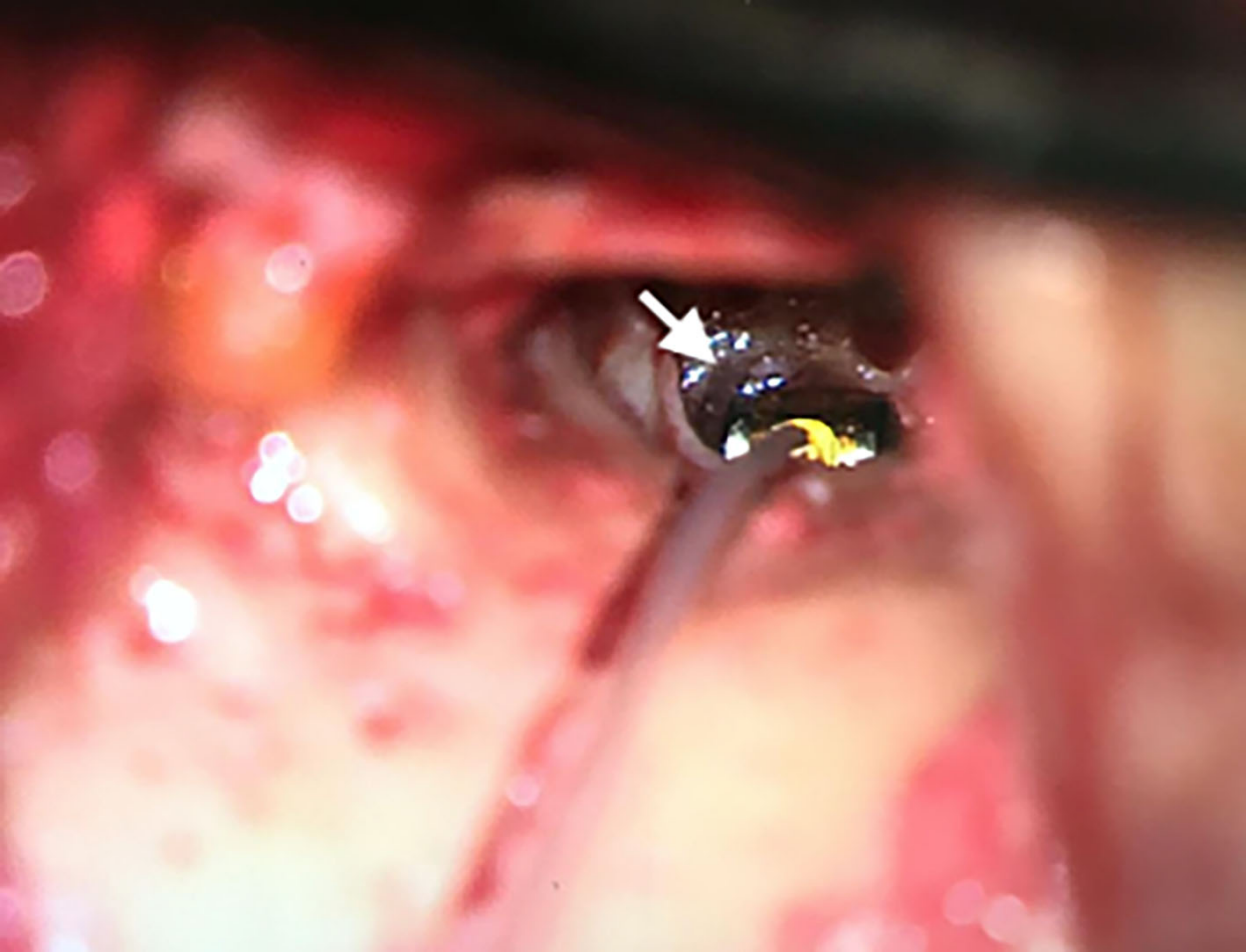
Transcanal identification of round window (white arrow), reshaping of the niche, and positioning of the floating mass transducer of the Vibrant Soundbridge.

With local-assisted anesthesia, we performed a transcanal approach with elevation of the tympanomeatal flap and preservation of the chorda tympani nerve with a minimally invasive retroauricular incision. Ossicular mobility and continuity were assessed, we excluded the stapedial fixation, and no cerebrospinal fluid (CSF) leak was observed during the surgical procedure. After identification and reshaping of the RW niche, a vibroplasty was performed paying particular attention to correctly plugging the round window and coupling it with the floating mass transducer (FMT) of the VSB (Figure 3). We opted to couple the FMT with the RW because concomitant RW plugging was performed and from previous studies it seemed to provide a more stable coupling over time than incus (8). No ossicular chain abnormalities or perilymphatic fistula were observed intraoperatively.

**Figure 4:**
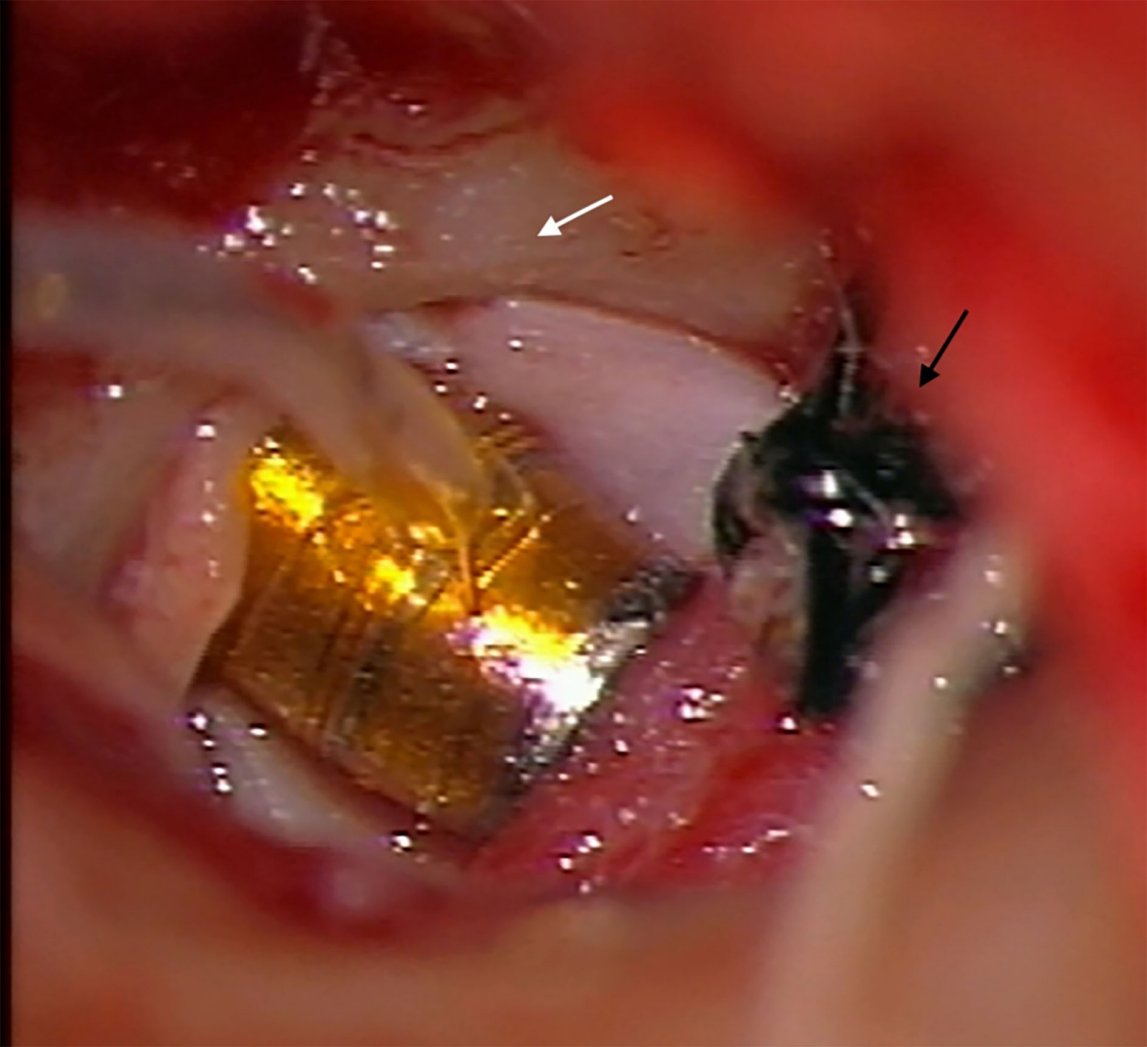
Plugging of the round window with FMT, cartilage, and perichondrium (tragus) (white arrow). These autologous tissues also helped to seal off the floating mass transducer in the round window niche. Intraoperatively hearing evaluation with RW electrocochleography (black arrow).

The plugging of the round window was achieved using cartilage and perichondrium (tragus). This autologous tissue also helped to seal off the FMT in the round window niche. Furthermore, VSB hearing outcomes were monitored with electrocochleography using a cotton-wick recording electrode placed on the hypotympanum (7) (Figure 4).

The wire of the VSB was housed in a canal tunnel drilled up to the tympanic attic. Minimal drilling of the cortical temporal bone posterosuperior to the external auditory meatus was necessary to house the implant receiver and extra wire (Figure 4). The ear canal tunnel was covered with autologous cartilage and external auditory meatus packing was performed.

This study received an exemption from the ethics committee of the University Hospital of Siena (Comitato Etico Regione Toscana, area vasta Sud Est–AOU Senese, Usl Toscana Sud Est) on 10/21/2019 for publication.

Surgery was uncomplicated, the patient did not complain of any post-operative vestibular symptoms. Sutures and external meatus packing were removed on the 10th postoperative day. At the 1-month follow-up, the patient underwent VSB activation and hearing and vestibular examination. She reported a significant improvement in auditory hypersensitivity and reduced sound distortion although tinnitus remained unchanged. No disabling vestibular symptoms were reported. Neither dizziness nor nystagmus could be observed in response to loud sounds or increased external ear pressure on the right side. The postoperative pure tone audiogram revealed a mild increase at 500, 1,000, and 2,000 Hz and a mild decrease at 250 and 4,000 Hz for bone conduction thresholds (Figure 1). An improvement to moderate hearing loss in the VSB-aided hearing threshold was confirmed at 3 months (Figure 1). The maximum speech recognition score of bysillabic words at 65 dB HL improved from 10% preoperatively to 70% at the last follow-up. The improvement of hearing and vestibular symptoms was confirmed subjectively by the patient on the right side. Discomfort and mild dizziness associated with loud sounds on the left side remained unchanged. Using a visual analog scale (0–10), the patient reported an improvement in symptoms from 10 to 4 and from 9 to 2, respectively, for hearing and vestibular complaints (3-month follow-up). Left side mild symptoms related to dehiscence remained unchanged. No short-term surgical complications such as device extrusion or external or middle ear canal infection/inflammation were identified at the 3-month follow-up (Table 1). Control HRCT was not performed since correct positioning of the FMT and plugging of the RW were confirmed by improvements in symptoms and stability of VSB-aided hearing. Air conduction VEMPs were not performed for safety reasons due to the risk of mobilizing the plugging or FMT from the RW.

## Discussion

To the best of our knowledge, this is the first report in the literature of local-assisted anesthesia with simultaneous RW reinforcement surgery and VSB positioning in a patient suffering from SSCD. The outcome of this new procedure confirmed the results in terms of safety and improvement in auditory and vestibular symptoms related to SSCD using the minimally invasive procedure for RW plugging reported by Silverstein et al. (6) and Succar et al. (9). The main new finding of this novel procedure is the possibility of using the VSB implant to improve the hearing threshold in patients with associated moderate to severe mixed hearing loss. The adverse effects of a traditional hearing aid fitting motivated us to adopt the vibroplasty procedure because the auditory gain is related to cochlear inner ear fluid movements that ideally do not determine vestibular end organ activation. The VSB-aided hearing threshold was significantly better than the preoperative value. Furthermore, the minimally invasive RW plugging and implantation procedure can be done with local anesthesia. When performed by experienced surgeons, there is minimal risk of iatrogenic sensorineural hearing loss.

The major improvements in terms of vestibular symptoms over auditory symptoms have been described in the literature for RW plugging vs. superior canal plugging. Several studies (5, 6, 9–11) have indicated that objective hearing outcomes are poorer with the transcanal RW plugging approach compared with canal resurfacing/plugging. In RW plugging, an increase in postoperative air conduction thresholds is common at lower frequencies due to the increased stiffness of the round window. Effects on higher frequencies are negligible because hydromechanical inertia and dissipative impedance of the cochlear fluids plays a major role. The association of VSB implantation with RW transcanal plugging can overcome this issue. The RW-aided gain is similar to that expected for this kind of procedure (7).

The main limitations of the present study are that it is a report on a single case, and there was only a short follow-up period.

In conclusion, simultaneous RW plugging and VSB positioning may be an effective, safe, and rapid surgical approach for SSCD associated with severe mixed hearing loss.

## Patient Perspective

Immediately after the surgical procedure the patient reported an improvement of vestibular symptoms due to loud sounds. She also reported a significant reduction in auditory hypersensitivity and sound distortion. Although tinnitus remained unchanged, the significant improvement in hearing threshold (VSB-aided) led to a higher quality of life.

## Data Availability Statement

The raw data supporting the conclusions of this article will be made available by the authors, without undue reservation.

## Ethics Statement

Written, informed consent, including all data and images, was obtained from the patient for the publication of this case report. Surgical procedure was performed in accordance with the Code of Ethics of the World Medical Association (Helsinki Declaration). This study received approval from the ethics committee of the Universitary Hospital of Siena (Comitato Etico Regione Toscana, area vasta Sud Est − AOU Senese, Usl Toscana Sud Est) for the publication on 10/21/2019.

## Author Contributions

MM performed the surgery. All authors contributed to the preparation and revision of manuscript and analyzed the data.

## Conflict of Interest

The authors declare that the research was conducted in the absence of any commercial or financial relationships that could be construed as a potential conflict of interest.
